# Author Correction: Large-scale control of the retroflection of the Labrador Current

**DOI:** 10.1038/s41467-023-43709-x

**Published:** 2023-11-29

**Authors:** Mathilde Jutras, Carolina O. Dufour, Alfonso Mucci, Lauryn C. Talbot

**Affiliations:** 1 Department of Earth and Planetary Sciences, McGill University, Montreal, QC Canada; 2 Department of Atmospheric and Oceanic Sciences, McGill University, Montreal, QC Canada; 3 Geotop, Université du Québec à Montréal, Montreal, QC Canada

**Keywords:** Physical oceanography, Physical oceanography

Correction to: *Nature Communications* 10.1038/s41467-023-38321-y, published online 06 May 2023

The original version of the Supplementary Information associated with this Article contained an error in Supplementary Figure S3, in which the panels for wind stress curl were inverted for strong and weak retroflection. The HTML has been updated to include a corrected version of the Supplementary Information.

### Supplementary information

Updated Supplementary Information

