# Supplementary material for: Large-scale control of the retroflection of the Labrador Current
Source: Nat Commun. 2023 May 6;14:2623. doi: 10.1038/s41467-023-38321-y (PMC10163246; doi:10.1038/s41467-023-38321-y)
Supplement: Supplementary file 1 — Supplementary information [file 41467_2023_38321_MOESM1_ESM.pdf]

Supplementary information to Large-scale control of the retroflection of the Labrador Current

Mathilde Jutras, Carolina O. Dufour, Alfonso Mucci, Lauryn C. Talbot

A Supplementary figures

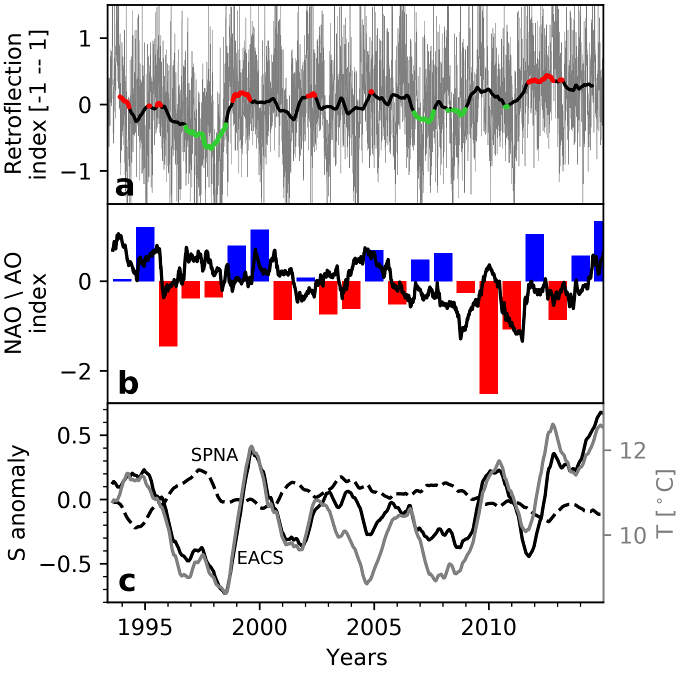

Figure S1: **(a)** Full (grey) and smoothed (black) retroflection index (see Fig. 3 of the main paper). **(b)** Indices of NAO (bars, bottom panel) and AO (black line, bottom panel). **(c)** Salinity in the subpolar North Atlantic (SPNA, dashed line) and temperature and salinity on the eastern American continental shelf (EACS, continuous lines), averaged over the top 500 m (boxes in Fig. S4a). Correlation coefficients with the retroflection index are respectively of -0.58, 0.57, and 0.57.

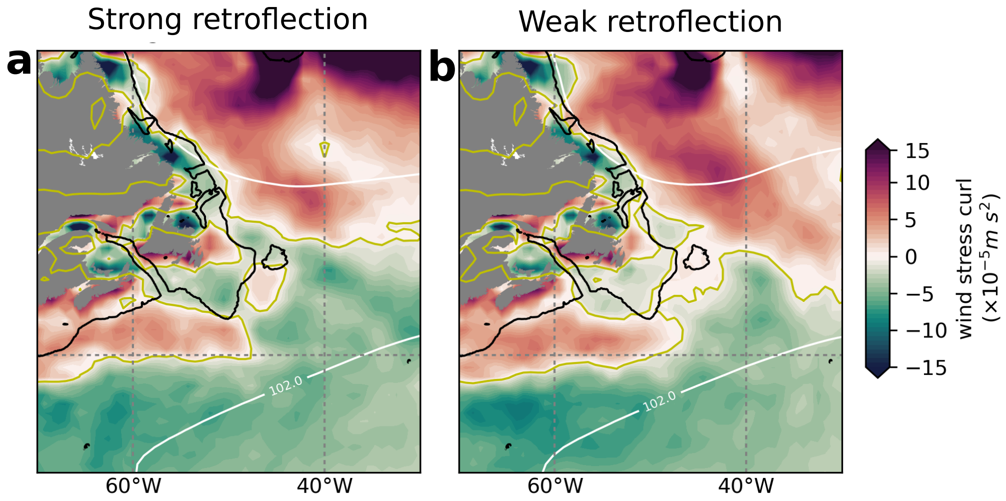

Figure S2: Composite maps of the wind stress curl. The yellow contours indicate the -0.5 wind stress curl lines, and highlight the connection between zones of positive wind stress curl during weak retroflection periods. The white contours are isolines of atmospheric pressure, in hPa. The black line delineates the 350 m isobath.

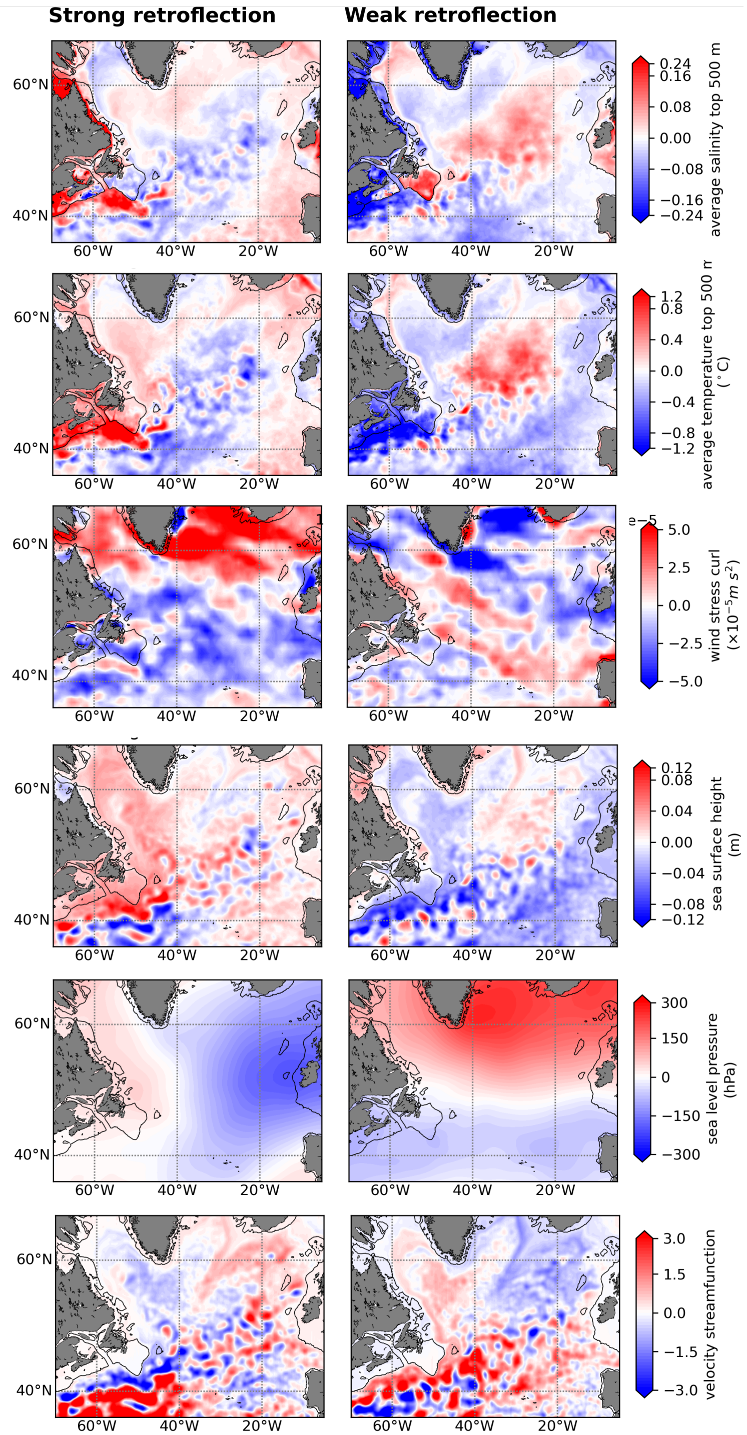

Figure S3: Composite maps in periods of strong (left) and weak (right) retroflection, for the anomalies of the same variables as Fig. 4. Composites are based on the periods identified in Fig. 3a.

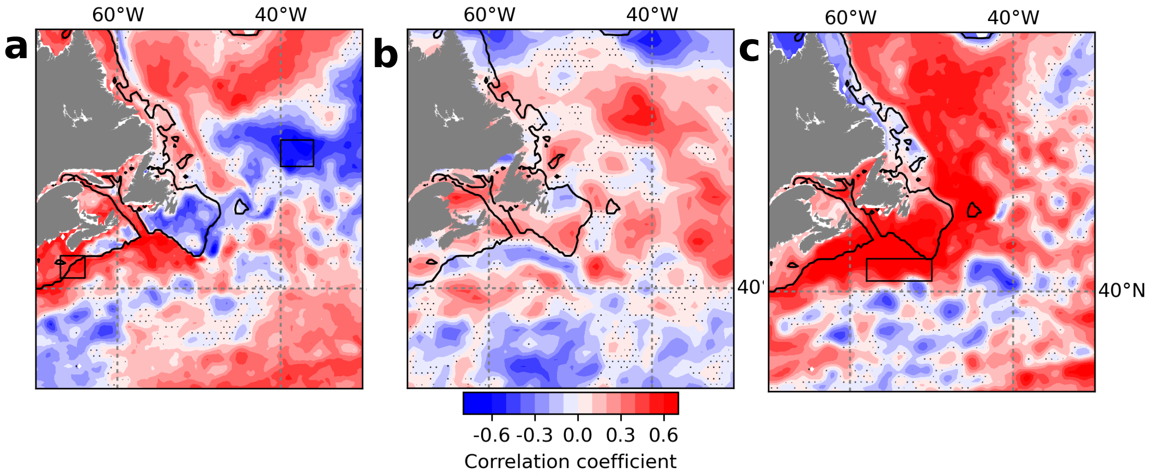

Figure S4: Correlation maps between the retroflection index and (a) average salinity in the top 500 m, (b) wind stress curl, and (c) sea surface height. The hatched zones indicate non-significant correlations (p>0.001). The boxes show zones of strong correlation used to compute the time series of Fig.S1 (boxes in a) and 3 (box in b).

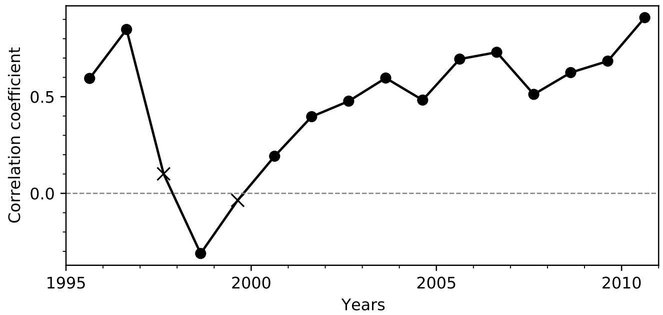

Figure S5: Correlation coefficient between the retroflection index and the volume transport of the Labrador Current, for 3-year segments. The dots indicate segments with significant correlations, and the crosses segments with non-significant correlations.

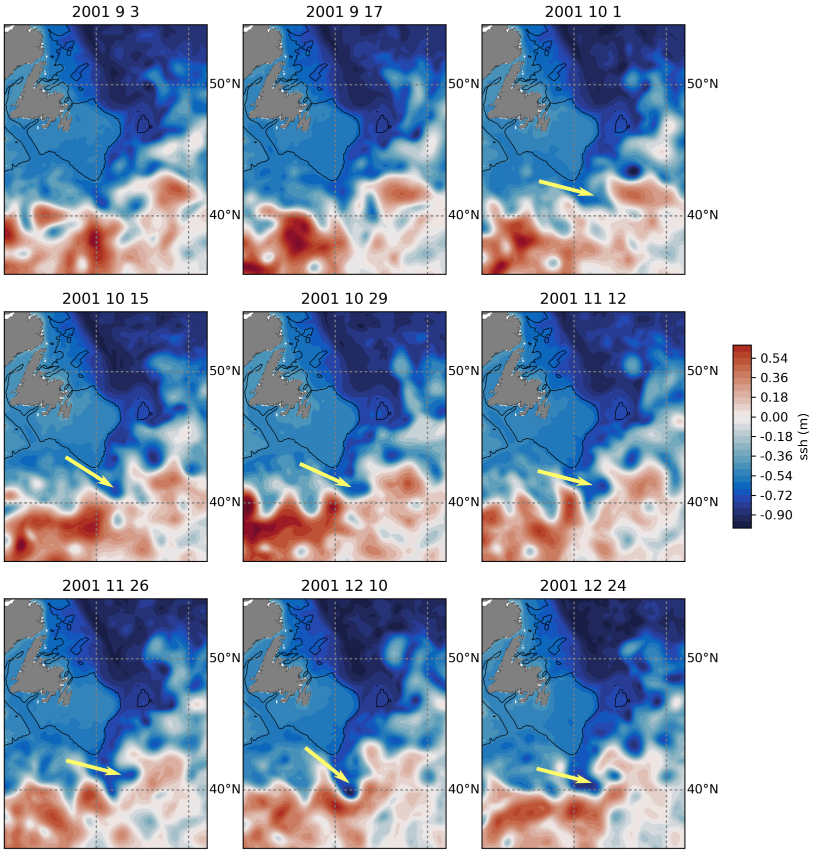

Figure S6: SSH maps showing the evolution of the formation of a cyclonic eddy (yellow arrow) at the tip of the Grand Banks from the tongue of Labrador Current Waters.

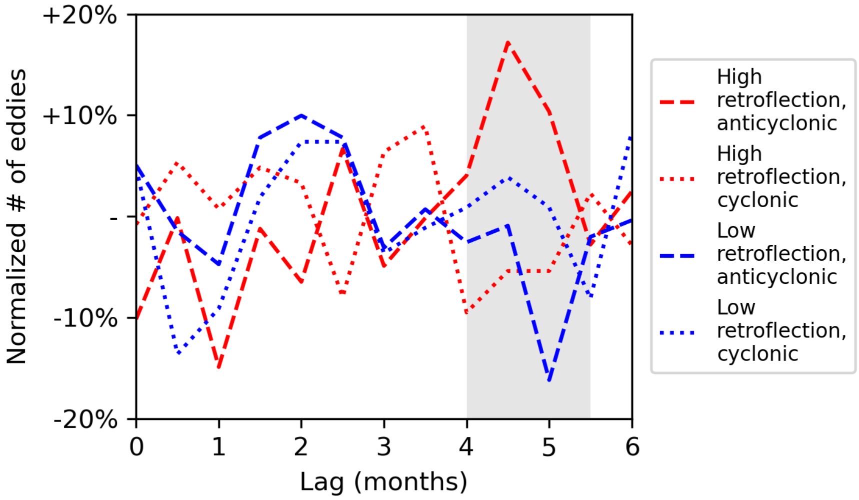

Figure S7: Anomalous number of eddies (in percentage) near the tip of the Grand Banks during strong (red) and weak (blue) retroflection periods based on the $\pm 1\sigma$ periods of the retroflection index, as a function of lag. We differentiate cyclonic (dotted) and anti-cyclonic (dashed) features. The grey band indicates the expected lag between the retroflection index and the time when eddies are counted.

B Supplementary information on the method

Retroflection index

A small number of virtual particles that leave the Labrador Current at the Grand Banks flow neither west towards the Slope Sea or the eastern American continental shelf, nor east towards the subpolar North Atlantic; rather, they go south from the tip of the Grand Banks. These represent approximately 7% of the particles, and their number shows little variability with time (Fig. S8). Since these particles do not affect the retroflection index significantly and for the sake of simplicity, we count them as retroflected.

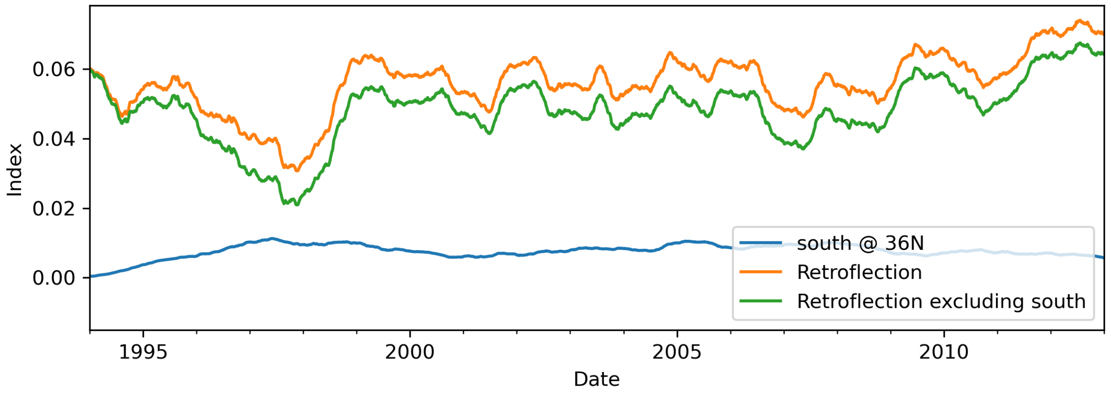

Figure S8: Time series of the number of particles going south from the Grand Banks (blue line), the retroflection index (orange line), and the retroflection index from which the particles going south are removed (green line).

In addition to the retroflection index based on Lagrangian trajectories, we compute a retroflection index based on an Eulerian approach. The Eulerian index is computed from the difference in the volume transport across the WB and SESPB sections (Fig. S14a). Whereas the Eulerian index represents the total transport, the Lagrangian index only tracks waters of subarctic origin. We therefore chose the Lagrangian index to study what controls the retroflection, but the two indices show a similar variability (not shown), and the Eulerian index is simpler to calculate.

Eliminated variables

During our analysis, we identified some variables that are not significantly correlated with the retroflection index. Whereas these results do not identify the forcing mechanisms at play, they help us understand what the main drivers are. The retroflection index has a low correlation with the freshwater transport. Furthermore, there is no clear relation between the retroflection index and the buoyancy fluxes, the mixed layer depths and convection along the shelf or in the Labrador Sea. This suggests that the dynamics of the system is the main determinant of the retroflection, while thermohaline processes play an indirect role, e.g. by affecting the strength of the transport. The

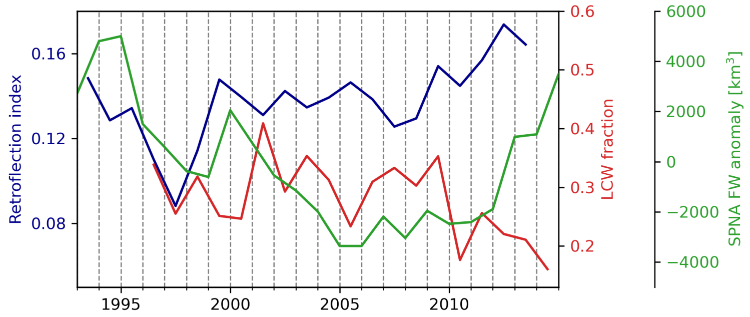

Figure S9: Yearly averave of the smoothed Lagrangian retroflection index (dark blue), of the observed contributions of the Labrador Current Waters (LCW, red) to the deep waters at the mouth of the Laurentian Channel, on the 1027.3 kg m^{-3} isopycnal [6], and of the freshwater content anomaly in the upper 1000 m of the subpolar North Atlantic (green), reproduced with authorization from [5].

buoyancy fluxes across the air-sea interface are calculated as follows:

$$B = -\rho \left[\alpha \frac{Q}{c_p} - \beta S(E - P) \right] \quad (1)$$

where α and β are, respectively, the thermal expansion and the haline contraction coefficients, both functions of salinity and temperature, Q is the air-sea heat flux, c_p is the heat capacity of water, and $(E - P)$ is the net freshwater flux from the ocean, or evaporation minus precipitation, neglecting the river run-off as we are far from the coasts [3].

C Validations

Validation of the ocean reanalysis

The GLORYS12V1 outputs are evaluated against measurements of temperature, salinity and velocity provided by the Department of Fisheries and Oceans (DFO) Canada along selected hydrographic sections [1, see Fig. S14a]. A visual inspection shows that the values, location and timing of the fronts, both associated with the Gulf Stream and that separating the Labrador Shelf from the Labrador Sea, are generally consistent with observations (Fig. S10). GLORYS12V1 reproduces the main features of the observed velocity field, including the structure and direction of jets on the continental shelf and the location of the shelf-break jet. It underestimates the velocity of the shelf-break jet at the latitude of the SI section, and the scarcity of the velocity data, with only one transect per summer, does not allow for a more quantitative estimate of this underestimation. The shelf-break jet is better resolved at lower latitudes, from the WB section downstream. Only the position of the NAC front at the SEGB section sometimes diverges from observations.

Comparison with trajectories of floats and drifters

The Lagrangian trajectories of virtual particles computed from the GLORYS12V1 ocean reanalysis are compared with trajectories of actual floats (Argo and RAFOS/SOFAR) and surface drifters.

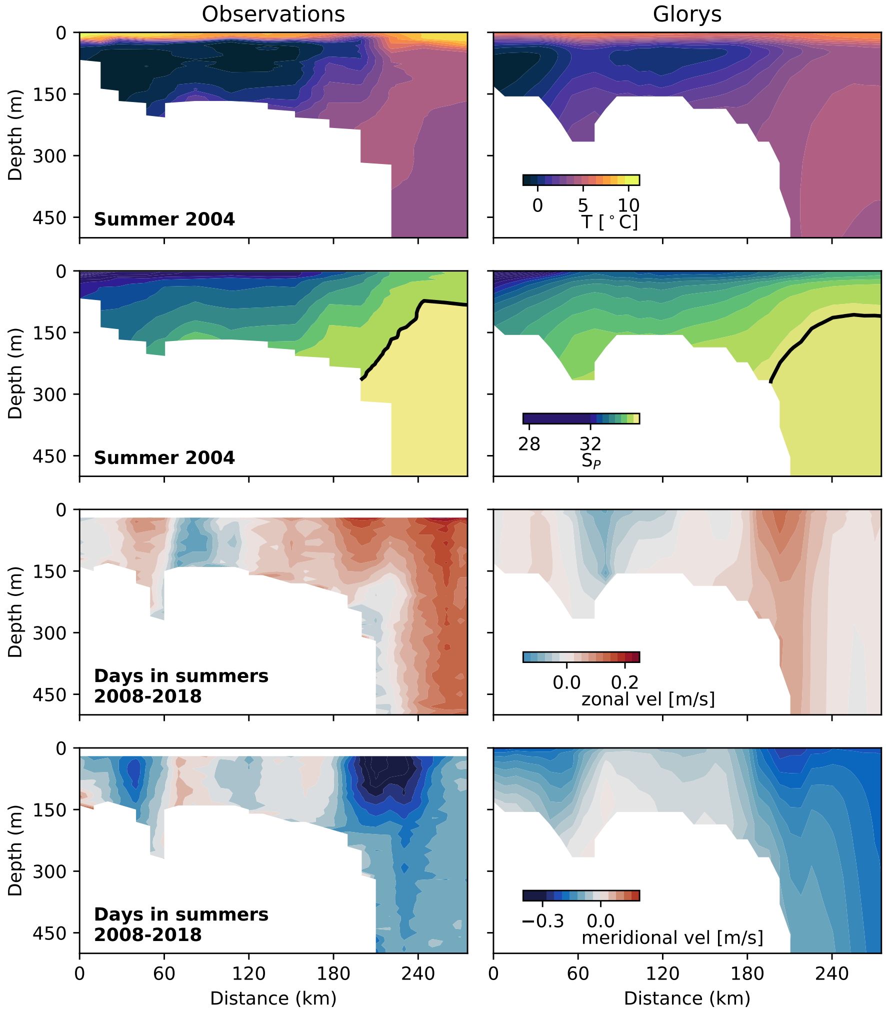

Figure S10: Comparison between DFO observations (left) and the GLORYS12V1 reanalysis (right) along the SI section (see Fig. S14a for the location of the hydrographic section). For temperature and salinity, observations from the 2004 sampling campaign are compared with the average values in summer 2004 in GLORYS12V1. For the zonal velocity and meridional velocity, we show the average over the sampled days, in summers of 2008–2018. The black contour indicates the 34.8 salinity cut-off used to discriminate the Labrador Shelf waters from the Labrador Sea.

There are two important differences to note between the model-based and the observation-based trajectories. (1) Most of the Argo and RAFOS/SOFAR floats drift deeper than our virtual particles, and are thus advected by the Deep Western Boundary Current (see section Method). (2) While

floats and drifters travel at a fixed depth, virtual particles can move vertically. Nevertheless, we use floats and drifters trajectories to perform a qualitative comparison with the virtual particle trajectories. A visual inspection suggests the pathways of the floats/drifters and of the virtual particles generally agree (Fig. 1b,c). Furthermore, similarly to the virtual particles, two major pathways emerge from the observational dataset in the area of the Grand Banks: a westward pathway into the Slope Sea, and an eastward (retroflecting) pathway towards the subpolar North Atlantic. The limited number of observational platforms drifting in the area and period of interest (193 in total, starting in 2000, Fig. S12) prevents us from performing a statistical analysis of the variability of the preferential pathway of these platforms. Nonetheless, we compare periods of weak retroflection (2003 and 2006-2007, Fig S11, middle) and strong retroflection (2009 and 2012-2016, Fig. S11, right) identified using the retroflection index. Since we only have observation-based trajectories starting from 2000 (Fig. S12), a comparison with the 1994-1996 weak retroflection period is impossible. In the periods of weak retroflection, 73 out of 90 floats and drifters (81%) retroflect, whereas 45 out of 49 (92%) retroflect during the periods of strong retroflection, qualitatively agreeing with what is found from the analysis of virtual particles.

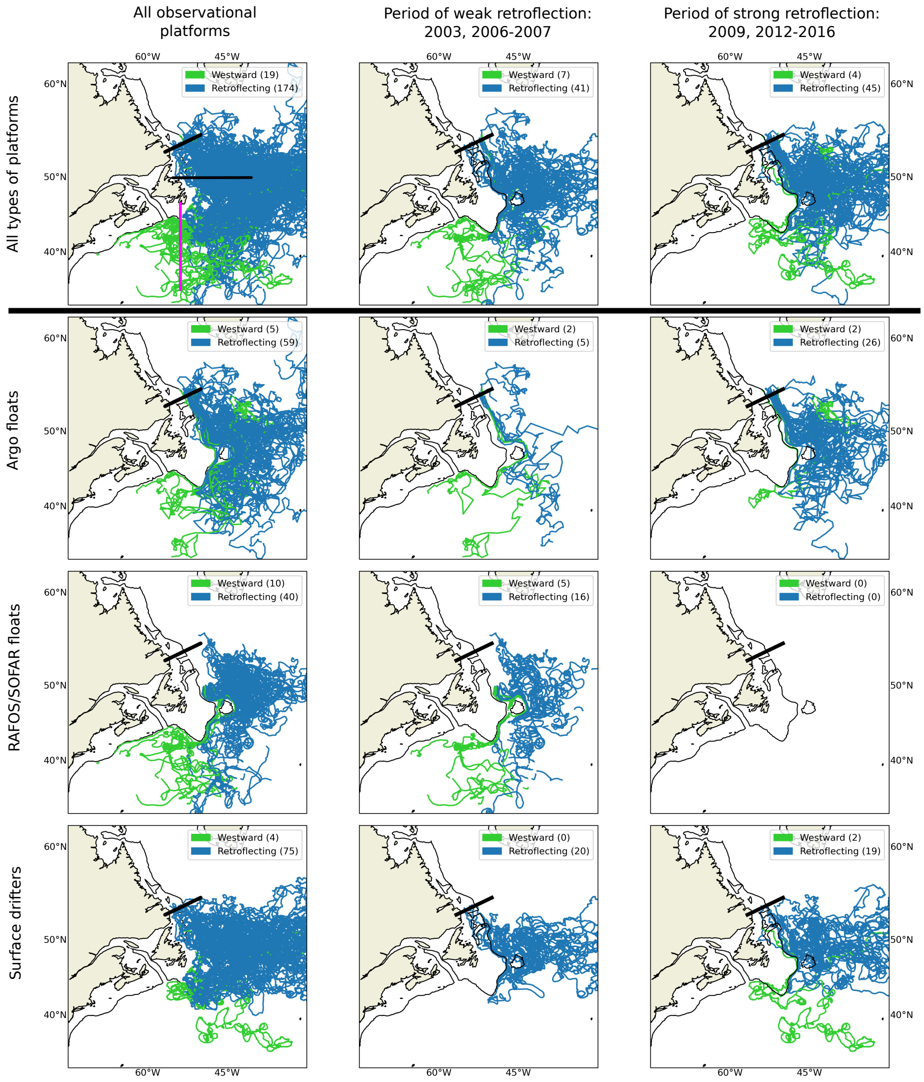

Figure S11: Observations from Argo floats (second row), RAFOS/SOFAR floats (third row) and surface drifters (bottom row) carried by the Labrador Current and passing through the Grand Banks region, in periods of weak (middle column) and strong (right column) retroflection identified from the retroflection index (see Fig. 3). The top row presents the three data types combined, and the left column the trajectories over the complete 2000-2018 period. The retroflected floats and drifters appear in blue, and the westward-flowing ones in green (see Fig. 1). The black and magenta lines in the first panel show the hydrographic line that the platforms have to cross to be considered. The black contour delineates the 350 m isobath.

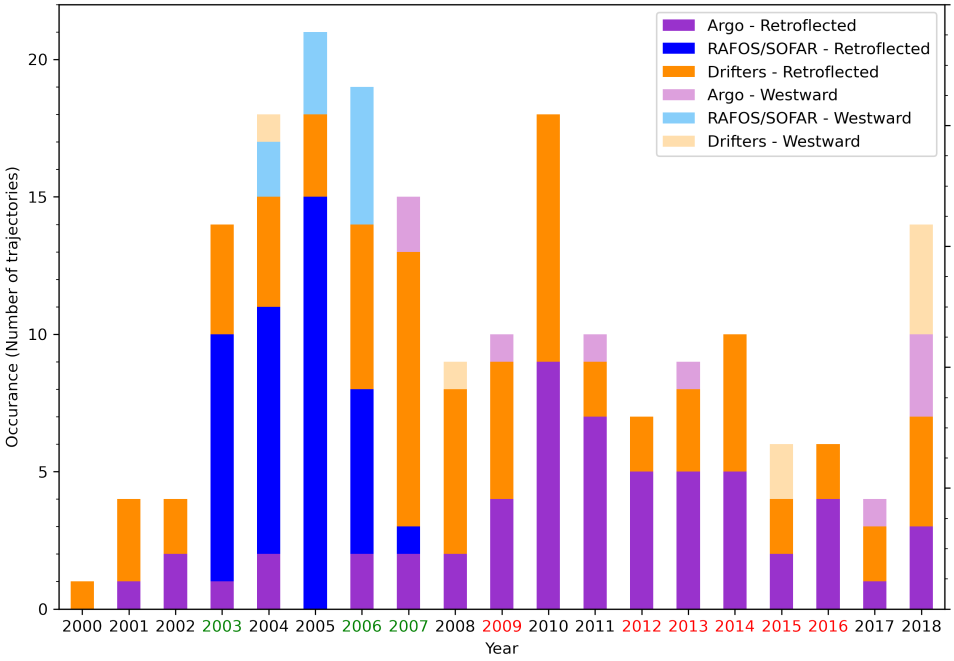

Figure S12: Distribution of floats and drifters over 2000–2018 categorized by float type: Argo floats (magenta), RAFOS and SOFAR floats (blue) and surface drifters (orange). The colour shading indicates if the floats retroflect (vivid) or move westward from the tip of the Grand Banks (dull). On the x-axis, years are identified as periods of weak (green) or strong (red) retroflection based on the retroflection index presented in Fig. 3.

D Labrador Current transport and circulation

Eulerian transport

In this section, we diagnose the volume transport and variability of the Labrador Current in the GLORYS12V1 reanalysis, and the coherence along its pathways. We compute the horizontal volume transport across various hydrographic sections monitored by DFO Canada on the Labrador Shelf, namely Seal Island (SI), White Bay (WB), Bonavista Bay (BB), Flemish Cap (FC), South-East Grand Banks (SEGB), South-East of St-Pierre Bank (SESPB), and Halifax Line (HL, see Fig. S14a). The outer edge of the section is chosen to cover the shelf-break jet. We find that the volume transport is weakly sensitive to small variations in the section edges.

The mean volume transport of the Labrador Current is 1.3 ± 0.2 (minimum of 0.6, maximum of 2.0) Sv on the Labrador Shelf (at the SI section) and 6.8 ± 1.0 (4.4–9.7) Sv for the shelf-break jet (Fig. S13). These numbers compare well with previous estimates from [8] of 6.3–9.8 Sv for total transport. At Flemish Cap, most of the current flows through the Flemish Pass, and only one quarter goes east around the Cap. As we move downstream, the Labrador Current progressively looses water offshore, and the total volume transport diminishes progressively (Fig. S13). Once the current reaches the Scotian Shelf (HL section), the mean volume transport drops to 0.6 ± 0.1 (0.1-1.0) Sv on the shelf and 1.3 ± 1.0 (0.0–4.0) Sv on the shelf-break.

The volume transport varies with time (Fig. S13). All along the shelf, the variance is of $\sim$ 0.1-0.2 Sv for the shelf component and of $\sim$1 Sv for the shelf-break component. Most of the variability follows a $\sim$6-year cycle on the Labrador Shelf, and around a 4-year cycle on the Scotian Shelf (from spectral analysis, not shown). The variability becomes less important in the last 10 years of the time series (2008-2018). There is a significant negative trend in the volume transport of the shelf-break branch all along the shelf ($\sim$ -0.05 Sv yr^{-1}, Mann-Kendall test at 0.05 significance level), consistent with Fig. 3, and in the volume transport of the shelf branch south of the Flemish Cap ($\sim$ -0.01 Sv yr^{-1}).

We investigate the coherence of the different branches and their interactions by correlating the volume transport of each branch at different locations along its path. A strong lagged correlation suggests that the current is not modified significantly from one location to the other. The transport of the shelf-break branch is coherent all along the Labrador Shelf (correlations $\sim$0.9, $p<0.001$). The transport is anti-correlated north and south of the Grand Banks, as reported by [4] (Fig. 3b). The shelf branch of the Labrador Current is somewhat coherent (correlations $\sim$0.5, $p<0.001$) on the Labrador Shelf and strongly coherent from Flemish Cap to the Scotian Shelf. The correlation is significant between the shelf and shelf-break branches in the Grand Banks area and southward, which suggests that there are significant interactions between the two branches in these area. A summary of these results is illustrated in Fig. S14a.

External contribution to the Labrador Current

As the Labrador Current flows southward from subarctic regions, it receives freshwater discharge from the continent and exchanges water with the Labrador Sea and the NAC. Since computing the volume transport from velocities does not keep track of the origin of the water, by comparing the volume transport derived from the Eulerian framework and the one reconstructed from the volume carried by Lagrangian particles, we can estimate the "external" contributions to the Labrador Current. We find that these contributions are of the order of 2 Sv all along the shelf, with slight variations depending on the location (Fig. S14b). The subarctic waters' contribution

to the Labrador Current diminishes dramatically as it flows south, reaching a few percents only on the Scotian Shelf. Given our interest in the fate of the fresh and well-oxygenated Labrador Shelf waters, the Lagrangian retroflection index is therefore more appropriate than the Eulerian index, as the latter contains the contributions from external sources.

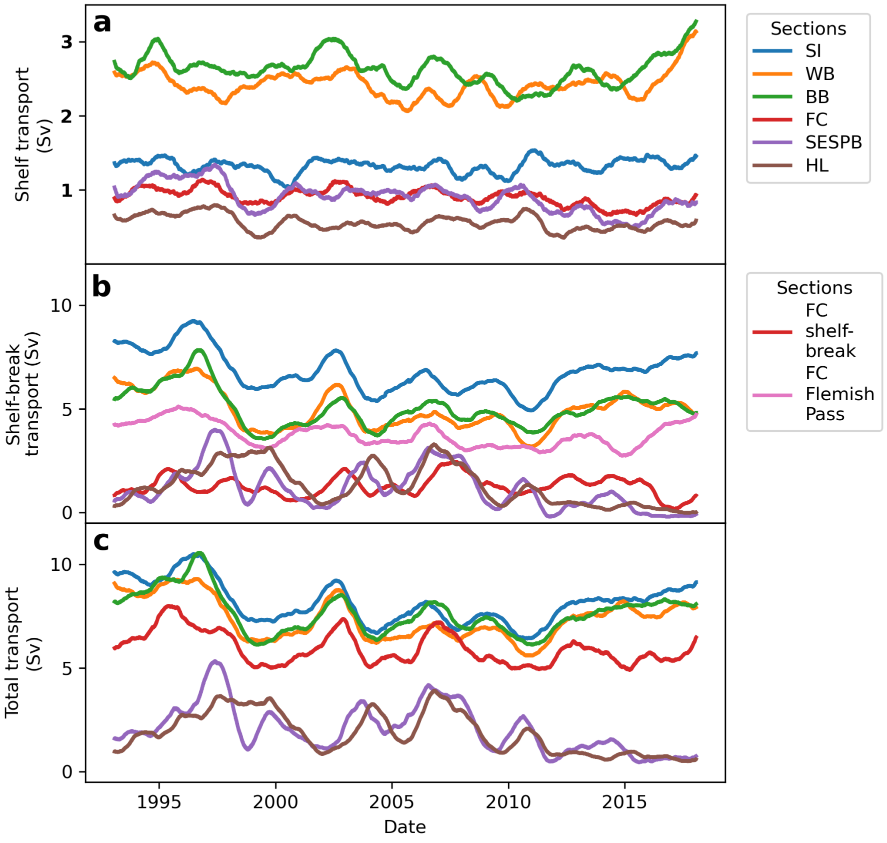

Figure S13: Time series of Labrador Current volume transport, computed from an Eulerian approach, on the shelf (a), at the shelf-break (b), and over both (c), at different sections, smoothed with a one-year moving average. For the location of the sections and the separation point between the shelf and shelf-break branches, see Fig. S14a.

Lagrangian trajectories

Whereas this paper focuses on the mechanisms leading to the retroflection of the Labrador Current, the Lagrangian trajectories we computed provide some interesting insights into the circulation of the area. Many of the particles seeded at intermediate depths (50-100 m) are upwelled to the

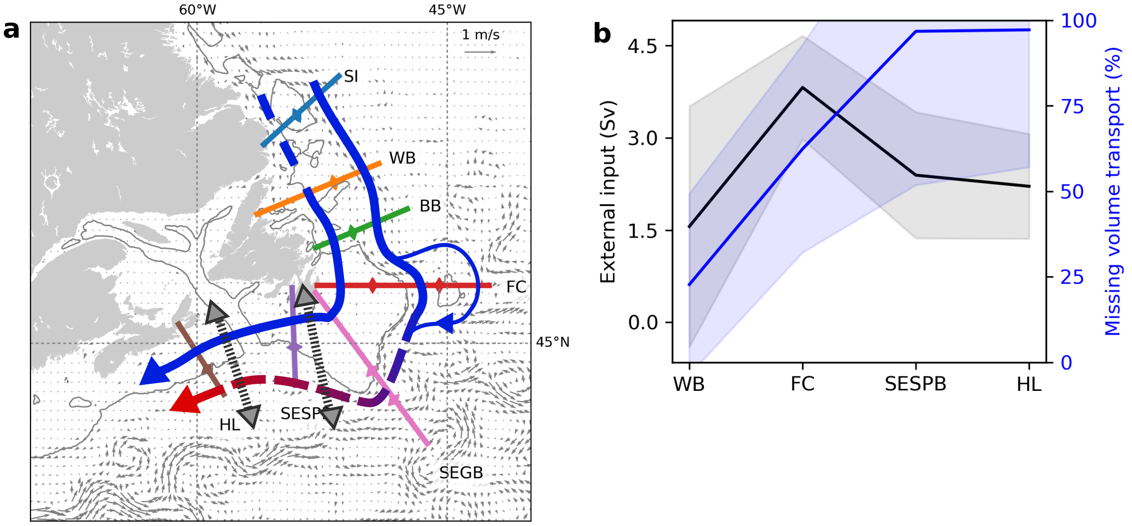

Figure S14: **(a)** Coherence within and interactions between branches of the Labrador Current. A full line indicates a high coherence (strongly correlated with its upstream self), and a dashed line some coherence. The color indicates the sign of the correlation (blue: positive, red: negative). The sign switch along the shelf-break branch indicates that the transport along the Scotian Shelf is anti-correlated with that along the Labrador Shelf. The fine dashed grey lines indicate locations of interactions between the shelf and shelf-break branches. We indicate the hydrographic sections (straight lines) and the separation points (diamonds) between the shelf and the shelf-break. The light grey arrows in the background provide a snapshot of the current speed averaged over the top 1000 m, or down to the seafloor if the waters are shallower than 1000 m. **(b)** In black: "external" contributions (not originating from the subarctic) to the Labrador Current, computed from the difference between the Eulerian and Lagrangian volume transport, at different sections. In blue: cumulative contribution (in percentage) of "external" contributions to the total volume transport across each section. The shaded area shows the standard deviation associated with the time series variability.

surface layer by the time they reach Flemish Cap. Most of the particles take 0-1 month to reach the WB section, 2-3 months to reach the FC section, 4-7 months to reach the SESPB section, and 8-15 months (a larger spread) to reach the HL section. These times equate to an advection velocity along the Labrador shelf of ~7-11 cm s^{-1}. In comparison, [7] measured velocities of 6-23 cm s^{-1} in the Labrador Current, and [2] and [9] found an advection velocity of ~20 cm s^{-1} by tracking salinity anomalies.

Supplementary References

[1] Cyr, F., E. Colbourne, P. S. Galbraith, O. Gibb, S. Snook, C. Bishop, N. Chen, G. Han, and D. Senciall, Physical Oceanographic Conditions on the Newfoundland and Labrador Shelf during 2018, *DFO Can.Sci. Advis. Sec. Res. Doc.*, p. 48 pp., 2020.

[2] Fratantoni, P. S., and M. S. McCartney, Freshwater export from the Labrador Current to the North Atlantic Current at the Tail of the Grand Banks of Newfoundland, *Deep-Sea Research Part I: Oceanographic Research Papers*, *57*(2), 258–283, doi:10.1016/j.dsr.2009.11.006, publisher: Elsevier, 2010.

[3] Gill, A. E., Transfer of Properties between Atmosphere and Ocean, in *Atmosphere—Ocean Dynamics*, vol. 30, pp. 19–38, Academic Press, doi:10.1016/S0074-6142(08)60027-3, iSSN: 00746142, 1982.

[4] Han, G., N. Chen, and Z. Ma, Is there a north-south phase shift in the surface Labrador Current transport on the interannual-to-decadal scale?, *Journal of Geophysical Research: Oceans*, *119*(1), 276–287, doi:10.1002/2013JC009102, iSBN: 2169-9275, 2014.

[5] Holliday, N. P., et al., Ocean circulation causes the largest freshening event for 120 years in eastern subpolar North Atlantic, *Nature Communications*, *11*(1), doi:10.1038/s41467-020-14474-y, iSBN: 4146702014474, 2020.

[6] Jutras, M., C. O. Dufour, A. Mucci, F. Cyr, and D. Gilbert, Temporal Changes in the Causes of the Observed Oxygen Decline in the St. Lawrence Estuary, *Journal of Geophysical Research: Oceans*, *125*(12), 1–20, doi:10.1029/2020JC016577, 2020.

[7] Lazier, J. R. N., and D. G. Wright, Annual velocity variations in the Labrador Current, *Journal of Physical Oceanography*, *23*(4), 659–678, doi:10.1175/1520-0485(1993)023<0659:AVVITL>2.0.CO;2, 1993.

[8] Petrie, B. D., and J. Buckley, Volume and freshwater transport of the Labrador Current in Flemish Pass, *Journal of Geophysical Research C: Oceans*, *101*(C12), 28,335–28,342, doi:10.1029/96JC02779, 1996.

[9] Petrie, B. D., S. Akenhead, and J. R. N. Lazier, Temperature and salinity variability on the eastern newfoundland shelf: The annual harmonic, *Atmosphere - Ocean*, *29*(1), 14–36, doi:10.1080/07055900.1991.9649390, 1991.
