# Supplementary material for: Large-scale control of the retroflection of the Labrador Current
Source: Nat Commun. 2023 May 6;14:2623. doi: 10.1038/s41467-023-38321-y (PMC10163246; doi:10.1038/s41467-023-38321-y)
Supplement: Supplementary file 3 — Description of Additional Supplementary Files [file 41467_2023_38321_MOESM3_ESM.pdf]

Description of Additional Supplementary Files

File Name: Supplementary Code 1

Description: Lagrangian tracking experiment in the Labrador Current using the OeanParcels Python package. Particles are seeded throughout the water column, along a hydrographic line crossing the Labrador Shelf. Kernels are added to OceanParcels that track the particles in 3 dimensions, remove stuck particles, particles exiting the domain of interest, and particles hitting the seafloor, as well as tracks additional properties of the water parcels including their age and volume.
